# Application of rapid in vitro co-culture system of macrophages and T-cell subsets to assess the immunogenicity of dogs vaccinated with live attenuated *Leishmania donovani* centrin deleted parasites (LdCen^−/−^)

**DOI:** 10.1186/s13071-016-1528-z

**Published:** 2016-04-30

**Authors:** Kelvinson Fernandes Viana, Jacqueline Araújo Fiuza, Sreenivas Gannavaram, Ranadhir Dey, Angamuthu Selvapandiyan, Daniella Castanheira Bartholomeu, Denise da Silveira-Lemos, Lilian Lacerda Bueno, Walderez Ornelas Dutra, Ricardo Toshio Fujiwara, Hira L. Nakhasi, Rodolfo Cordeiro Giunchetti

**Affiliations:** Laboratory of Cell-Cell Interactions, Morphology Department, Institute of Biological Science, Federal University of Minas Gerais, Av. Antônio Carlos, 6627, Pampulha, Belo Horizonte, Minas Gerais 31270-901 Brazil; Laboratory of Biomolecules and Vaccines, Agrarian Sciences and Technologic Department, Federal University of Tocantins, Gurupi, Tocantins Brazil; Laboratory of Cellular and Molecular Immunology, René Rachou Research Center, Oswaldo Cruz Foundation, Belo Horizonte, MG Brazil; Division of Emerging and Transfusion Transmitted Diseases, Office of Blood Research and Review, Center for Biologics Evaluation and Research, US Food and Drug Administration, 10903 New Hampshire Ave, Silver Spring, MD 20993 USA; Institute of Molecular Medicine, 254 Okhla Industrial Estate, Phase III, New Delhi, 110020 India; Laboratory of Immunology and Genome of Parasites, Parasitology Department, Institute of Biological Science, Federal University of Minas Gerais, Belo Horizonte, Minas Gerais Brazil; Laboratory of Diagnosis Biomarkers and Monitoring, René Rachou Research Center, Oswaldo Cruz Foundation, Belo Horizonte, Minas Gerais Brazil

**Keywords:** Vaccine, In vitro co-culture, CD4^+^ and CD8^+^ T-cells, Canine visceral leishmaniasis LdCen^−/−^, Leishmune®

## Abstract

**Background:**

Live attenuated *Leishmania donovani* parasites as LdCen^−/−^ were shown to confer protective immunity against *Leishmania* infection in mice, hamsters, and dogs. Strong immunogenicity in dogs vaccinated with LdCen^−/−^ has been previously reported, including increased antibody response favoring Th1 response lymphoproliferative responses, CD4^+^ and CD8^+^ T-cells activation, increased levels of Th1 and reduction of Th2 cytokines, in addition to a significant reduction in parasite burden after 18 and 24 months post virulent parasite challenge.

**Methods:**

Aimed at validating a new method using in vitro co-culture systems with macrophages and purified CD4^+^ or CD8^+^ or CD4^+^:CD8^+^ T-cells of immunized dogs with both LdCen^−/−^ and Leishmune® to assess microbicide capacity of macrophages and the immune response profile as the production of IFN-γ, TNF-α, IL-12, IL-4 and IL-10 cytokines.

**Results and discussion:**

Our data showed co-cultures of macrophages and purified T-cells from dogs immunized with LdCen^−/−^ and challenged with *L. infantum* were able to identify high microbicidal activity, especially in the co-culture using CD4^+^ T-cells, as compared to the Leishmune® group. Similarly, co-cultures with CD8^+^ T-cells or CD4^+^:CD8^+^ T-cells in both experimental groups were able to detect a reduction in the parasite burden in *L. infantum* infected macrophages. Moreover, co-cultures using CD4^+^ or CD8^+^ or CD4^+^:CD8^+^ T-cells from immunized dogs with both LdCen^−/−^ and Leishmune® were able to identify higher levels of IFN-γ and IL-12 cytokines, reduced levels of IL-4 and IL-10, and a higher IFN-γ/IL-10 ratio. While the highest IFN-γ levels and IFN-γ/IL-10 ratio were the hallmarks of LdCen^−/−^ group in the co-culture using CD4^+^ T-cells, resulting in strong reduction of parasitism, the Leishmune® immunization presented a differential production of TNF-α in the co-culture using CD4^+^:CD8^+^ T-cells.

**Conclusion:**

The distinct conditions of co-culture systems were validated and able to detect the induction of immune protection. The method described in this study applied a new, more accurate approach and was able to yield laboratory parameters useful to test and monitor the immunogenicity and efficacy of *Leishmania* vaccines in dogs.

## Background

Leishmaniasis is a protozoonosis that is endemic in several countries around the world, with more than 350 million cases being visceral leishmaniasis [1]. The impact of controlling the infected dog population to reduce human visceral leishmaniasis prevalence in these endemic areas has been debated [2–4]. Therefore, the development of a canine visceral leishmaniasis (CVL) vaccine is highly desirable and would represent the most practical and efficient control tool [5].

A key goal in the control of CVL has been the development of vaccines with high protective capability to interrupt the parasite transmission [6]. Assessments of vaccine safety and anti-CVL efficacy generally require a long follow-up, extending into years of study [7–9]. Thus, researchers need to develop methodological strategies that enable a more rapid and accurate evaluation of the dog’s immune system. Such tests could be included in clinical trials of vaccines against CVL, so that the time needed for the experiments could be reduced. This would likely reduce the costs of experimentation using the dog model and provide a more rational way of selecting candidate vaccines against CVL.

Among the live-attenuated parasites used as vaccines, the centrin deletion in *L. donovani* (LdCen^−/−^) has been shown to specifically affect the cytokinesis and lead to multinucleated cells and eventual cell death of amastigote forms while the growth of promastigote forms is unaffected. Previous studies evaluated the protective immunity of LdCen^−/−^ and demonstrated the safety, immunogenicity, and protection against infection with wild type *L. donovani* in mice and hamster models [10]. Recently, we have demonstrated that immunization with LdCen^−/−^ results in an increase in immunoglobulin isotypes, higher lymphoproliferative response, higher frequencies of activated CD4^+^ and CD8^+^ T-cells, IFN-γ production by CD8^+^ T-cells, increased secretion of TNF-α and IL-12, and decreased secretion of IL-4, as well as a significant reduction in parasite burden 18 and 24 months after experimental challenge in dogs [11].

Recently, an in vitro co-culture system using macrophages and purified T-cells was standardized to analyze the adaptive immune response in dogs immunized against CVL [12]. In this study, we applied the same method using LdCen^−/−^ and Leishmune® vaccines, which have been reported to induce a strong immune response against canine visceral leishmaniasis. The microbicidal capability of macrophages co-cultured with CD4^+^ or CD8^+^ T-cells or CD4^+^:CD8^+^ T-cells simultaneously was assessed after in vitro infection with *L. infantum* and the cytokine levels were analyzed from dogs after 24 months of the experimental challenge with *L. infantum*. The results suggest that this in vitro co-culture system allows rapid efficacy analysis of vaccines against canine visceral leishmaniasis.

## Methods

### Parasites

The wild type strain of *L. infantum* (MHOM/BR/1970/BH46) was used for the in vitro co-culture system. The parasites were grown in NNN/LIT (Sigma Chemical Co., USA) culture medium supplemented with inactivated 20 % fetal bovine serum (FBS) (Cultilab, Brazil), plus penicillin (200 U/ml) and streptomycin (100 μg/ml), at pH 7.4 and incubation temperature of 23 °C. Parasites used for in vitro tests were removed from the culture at the stationary phase (seventh day of culture) during the seventh passage. Parasites were counted in a Neubauer chamber, from which more than 90 % viability was obtained and later used for in vitro infection.

### Animals and vaccination protocol

Eighteen healthy beagle dogs with 8 months of age were divided into three groups (three males and three females per group). The *L. donovani* centrin-deleted (LdCen^−/−^) parasites were used for immunization. The LdCen^−/−^ group received 10^7^ LdCen^−/−^ stationary phase promastigotes subcutaneously (single-dose). The Leishmune® group received three subcutaneous doses of vaccine (1 ml each) with an interval of 21 days between each dose, as recommended by the manufacturer (Zoetis, Brazil). The Control group received PBS alone. All animals were challenged 2 months after the last dose of vaccine (Leishmune® or LdCen^−/−^) or PBS. The blood collection to analyze the co-culture systems was performed 24 months after an intravenous challenge with 10^7^ of stationary phase promastigotes of *L. infantum*.

#### Ethical approval

The study was approved by the Ethical Committee for Use of Experimental Animals of the Federal University of Minas Gerais, Brazil (CETEA Protocol no.122/09 and 288/2012).

### Isolation of peripheral blood mononuclear cells

Blood samples (10 ml each) from the 17 dogs (control group: *n* = 6; Leishmune® group: *n* = 5; LdCen^−/−^ group: *n* = 6) were collected in heparinized tubes intended for obtaining peripheral blood mononuclear cells (PBMC), as previously described [13]. Briefly, the whole blood volume collected was placed in a mixture of Ficoll-Hypaque (Sigma Chemical Co.; density: 1.119 g/ml) and Ficoll-Hypaque (Sigma Chemical Co.; density: 1.077 g/ml) at a 1:3 ratio (Ficoll/blood) in sterile polystyrene conical bottom tubes (Falcon, Corning, USA). All samples were centrifuged at 700 × *g* for 80 min at 22 °C. The PBMC ring was collected at the Ficoll-Hypaque interface and transferred to another tube with 40 ml of Falcon sterile 1× PBS containing 10 % FBS. This tube was centrifuged twice at 400 × *g* for 10 min at 4 °C. After the supernatant was discarded, the cells were resuspended in 1 ml of cell culture medium RPMI 1640. Cells were counted in a Neubauer hemocytometer chamber to determine the numbers of monocytes or lymphocytes per milliliter.

### Microbicidal activity

The microbicidal activity was assessed using monocytes, which were adjusted and plated at 5 × 10^5^ monocytes/well using 24-well plates (NUNC, Thermo Fisher Scientific Inc., USA), on circular coverslips (15 mm; Glasscyto, Brazil), as previously described [13]. Briefly, cultures were established using RPMI supplemented with 20 % fetal calf serum on 20 % macrophage colony-stimulating factor medium obtained from supernatant of cultures of L929 immortalized cells, and incubated at 37 °C/5 % CO_2_. Monocytes differentiating into macrophages were evaluated at 5 days of culture, as previously described [12, 13]. The cells were infected with 5 × 10^6^ of *L. infantum* promastigotes in the stationary phase, using a 10:1 ratio (ten parasites per macrophage). Each well was gently washed 3 h after infection, and the cultures were maintained in co-culture with CD4^+^ or CD8^+^ T-cells or CD4^+^:CD8^+^ T-cells simultaneously to assess microbicidal activity at 72 h after infection. To calculate the rate of parasitic infection, the numbers of amastigotes in 200 macrophages were counted. Afterwards, the total number of amastigotes was divided by the total number of infected macrophages to obtain the average number of amastigotes per macrophage [12, 13].

### Purification of CD4^+^ and CD8^+^ T-cells

After obtaining the PBMC by Ficoll-Hypaque gradient, lymphocytes were submitted to further purification, as previously described [12, 13]. CD4^+^ and CD8^+^ T-lymphocytes were isolated using magnetic beads (Miltenyi Biotec Inc., USA) by positive selection using anti-CD4 or anti-CD8-FITC (fluorescein isothiocyanate) antibodies (AbD Serotec, UK) and microbeads coated with anti-FITC. Cell suspension was prepared at a concentration of 6 × 10^7^ cells in a 1-ml tube in an isolation buffer containing PBS 1×, pH 7.2, 0.5 % BSA, and 2 mM EDTA. Monoclonal antibodies (CD4 or CD8-FITC) were added to 2 μl/ml of total lymphocytes and incubated at room temperature (RT) for 15 min. Next, magnetic microbeads were added to 10 μl/ml lymphocytes and incubated for 15 min at RT. Cell suspension was loaded into a MACS column (Miltenyi Biotec Inc.), which was placed in the magnetic field of a MACS separator. The magnetically labeled CD4^+^ or CD8^+^ cells were retained in the column, whereas the unlabeled cells ran through, resulting in this cell fraction being depleted of CD4^+^ and CD8^+^ cells. After removing the column from the magnetic field, the magnetically retained CD4^+^ and CD8^+^ cells were eluted as the positively selected cell fraction by washing the magnetic column with 15 ml of isolation buffer.

The purity of CD4^+^ and CD8^+^ T-cells was evaluated by flow cytometry on a FACSCalibur instrument (Becton Dickinson, USA) interfaced to an Apple G3 workstation. Cell-Quest software (Becton Dickinson) was used for both data acquisition and analysis. A total of 20,000 events were acquired for each preparation. Flow cytometric analysis was performed using canine whole blood leukocytes that were selected on the basis of their characteristic forward (FSC) and side (SSC) light-scatter distributions. Following FSC and SSC gain adjustments, the lymphocytes were selected by gating 1 on the FSC versus SSC graph. Fluorescence was evaluated from FITC spectra (anti-CD4 and anti-CD8 antibodies) on FL1 in dot plot representations. A marker was set as an internal control for nonspecific binding to encompass > 98 % of the unlabeled cells, and this marker was then used to analyze data for individual animals. The results were expressed as the percentage of positive cells within the selected gate for T-cell surface markers presenting CD4^+^ or CD8^+^.

### Co-culture system

After purification, the CD4^+^ and/or CD8^+^ T-cells were incubated with macrophage (Mac) previously infected with *L. infantum* promastigotes (10 parasites per macrophage) in the following proportions: to CD4^+^ T-cells: Mac - 1 lymphocyte for each 2 macrophages (1:2) and to CD8^+^ T-cells: ac - 1 lymphocyte for each 2 macrophages (1:2). The culture of CD4^+^: CD8^+^ T-cells simultaneously incubated under the same condition used ratios of 1:1:1 (CD4^+^: CD8^+^: Mac). Cells were grown in this co-culture system for 72 h, as previously described [12, 13]. Afterwards, the well supernatants were collected and stored at -80 °C for later cytokine measurements.

### ELISA cytokine assay

Culture supernatants were collected at 72 h, and the levels of IFN-γ, TNF-α, IL-12, IL-4, and IL-10 were titrated in culture supernatants using the ELISA method (R&D Systems, USA). The plates were coated with anti-IFN-γ, anti-TNF-α, anti-IL-12, anti-IL-4, and anti-IL-10 mAb in PBS, pH 7.4, and incubated at 4 °C overnight. After blocking the wells using buffer containing PBS plus 0.05 % (*v/v*) Tween 20 and 0.1 % (*w/v*) BSA, supernatants were added to each well. Biotin-labeled mAb in an incubation buffer was added to each well, and streptavidin-HRP was used as an enzyme. The reaction was developed using 3,3′,5,5′-tetramethylbenzidine substrate and stopped by adding a 2.5 M H_2_SO_4_ solution. The plates were washed after each step using PBS plus 0.05 % (*v/v*) Tween 20. Minimum sensitivity levels were 119 pg/ml for IFN-γ, 109 pg/ml for TNF-α, 102 pg/ml for IL-12, 118,08 pg/ml for IL-4, and 89 pg/ml for IL-10. All experiments were performed using 96-well plates (COSTAR, Corning Inc., USA) and according to R&D Systems instructions. Reading was performed using a microplate automatic reader (EL800; Biotek, Winooski, VT, USA) at a wavelength of 450 nm.

### Statistical analysis

Statistical analysis was performed with GraphPad Prism 5.0 software (Prism Software, CA, USA). Data normality was assessed using the Kolmogorov-Smirnov test. The analyses of co-cultures and cytokines were performed using repeated ANOVA measurements. Differences were considered significant at *P* < 0.05.

## Results

### Immunization with LdCen^−/−^ attenuated parasites resulted in significant microbicidal activity of macrophages co-cultured with CD4^+^ T-cells associated with increased levels of Th1 cytokines

**Fig. 1:**
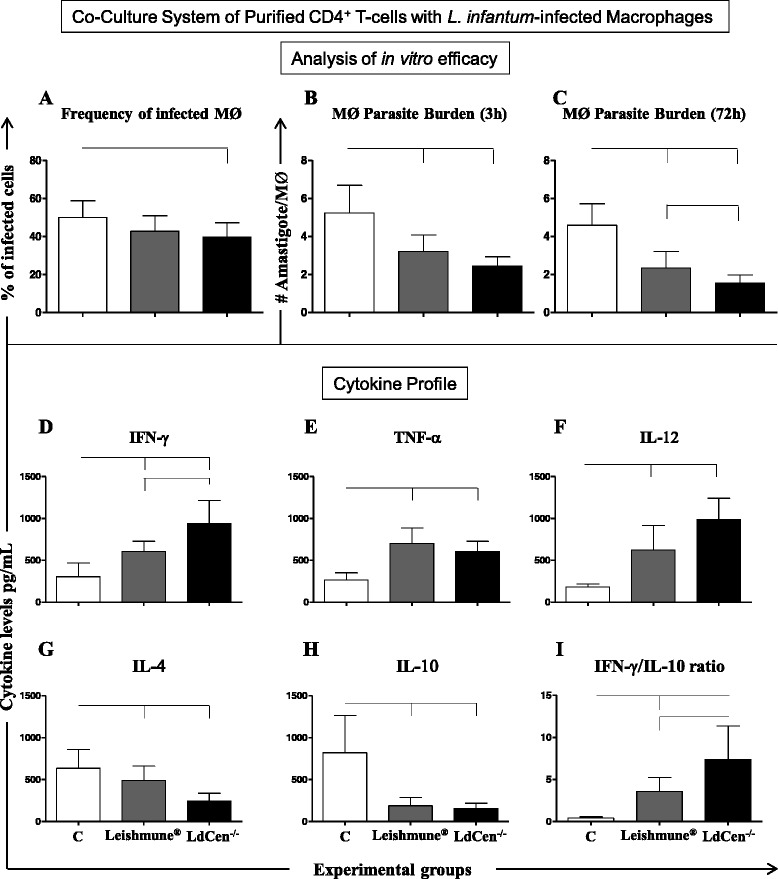
Co-culture system using *L. infantum* infected macrophages (MØ) and CD4^+^ T-cells, 24 months after experimental challenge with *L. infantum*. Control group (*n* = 6 dogs) and Leishmune® (*n* = 5 dogs) received three doses of PBS or vaccine, respectively, at 21 day intervals; LdCen^−/−^ group (*n* = 6 dogs) received 10^7^ LdCen^−/−^ stationary phase promastigotes in single-dose. **a** Infection rate (%) of macrophages after 3 h of in vitro infection with *L. infantum* (5 × 10^6^ promastigotes; ratio ten parasites: 1 MØ). **b** Number of amastigote forms by macrophages after 3 h of in vitro infection with *L. infantum*. **c** Number of amastigote forms by macrophages after 72 h of in vitro infection with *L. infantum*. The cytokine profile (pg/mL) was analyzed in supernatant from *L. infantum* infected macrophages (MØ) co-cultured with CD4^+^ T-cells for IFN-γ (**d**), TNF-α (**e**), IL-12 (**f**), IL-4 (**g**), IL-10 (**h**) and IFN-γ/IL-10 ratio (**i**)*.* The *x*-axis represents the control group (C), Leishmune® and LdCen^−/−^ vaccines. The connecting lines between the bars indicate significant differences (*P* < 0.05)

The performance of the co-culture system was evaluated, including CD4^+^ T-cells, to analyze the contribution of this cell subpopulation in the immunogenicity establishment. After 3 h of in vitro infection of macrophages with *L. infantum*, macrophages from the LdCen^−/−^ group showed a lower (*P =* 0.049) frequency of infection (Fig. 1a) as compared to the control group. The parasite burden was reduced in macrophages in both LdCen^−/−^ (*P* = 0.0009) and Leishmune® (*P* = 0.005) groups as compared to the control (Fig. 1b). Interestingly, 72 h after in vitro infection, LdCen^−/−^ group displayed an intense reduction (*P* = 0.012) in parasite burden as compared to the Leishmune® group (Fig. 1c). LdCen^−/−^ (*P* = 0.008) and Leishmune® (*P* = 0.005) immunizations induced high levels of cytokines IFN-γ (Fig. 1d), TNF-α (Fig. 1e) and IL-12 (Fig. 1f) and low levels of IL-4 (Fig. 1g) and IL-10 (Fig. 1h) in co-cultures as compared to the control group. Remarkably, the LdCen^−/−^ presented high levels of IFN-γ when compared to the Leishmune® group (Fig. 1d). Furthermore, both analyzed vaccines presented a high IFN-γ/IL-10 ratio as compared to the control group, with LdCen^−/−^ displaying the highest (*P* = 0.006) cytokine ratio (Fig. 1i).

### LdCen^−/−^ and Leishmune® immunized dogs induced an intense microbicidal activity of macrophages co-cultured with CD8^+^ T-cells and increased levels of pro-inflammatory cytokines

**Fig. 2:**
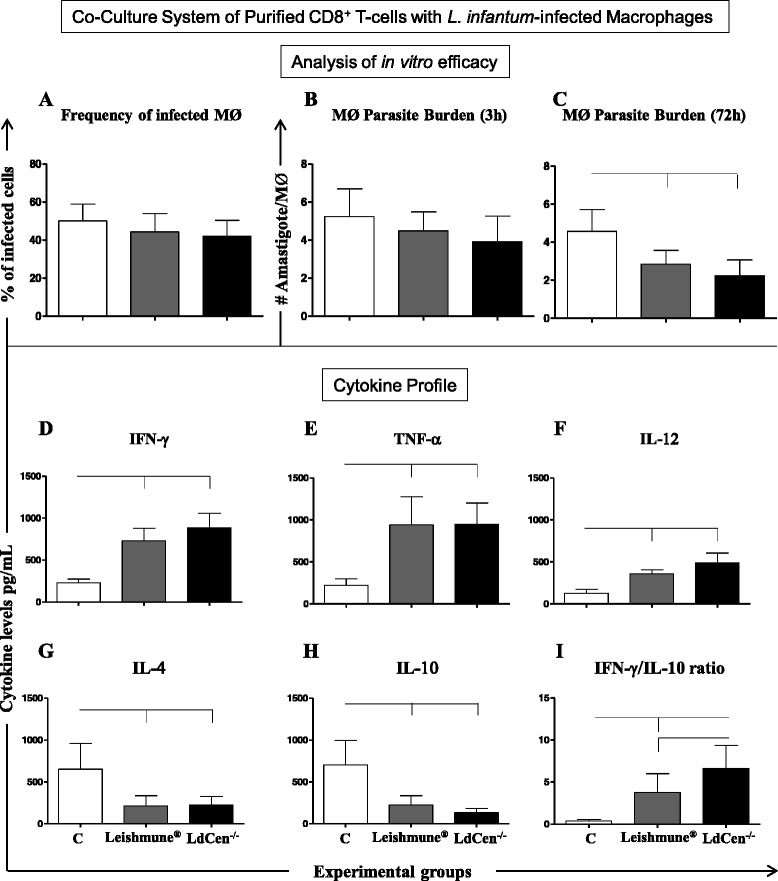
Co-culture system using *L. infantum* infected macrophages (MØ) and CD8^+^ T-cells, 24 months after experimental challenge with *L. infantum*. Control group (*n* = 6 dogs) and Leishmune® (*n* = 5 dogs) received three doses of PBS or vaccine, respectively, at 21 day intervals; LdCen^−/−^ group (*n* = 6 dogs) received 10^7^ LdCen^−/−^ stationary phase promastigotes in single-dose. **a** Infection rate (%) of macrophages after 3 h of in vitro infection with *L. infantum* (5 × 10^6^ promastigotes; ratio ten parasites: 1 MØ). **b** Number of amastigote forms by macrophages after 3 h of in vitro infection with *L. infantum*. **c** Number of amastigote forms by macrophages after 72 h of in vitro infection with *L. infantum*. The cytokine profile (pg/mL) was analyzed in supernatant from *L. infantum* infected macrophages (MØ) co-cultured with CD8^+^ T-cells for IFN-γ (**d**), TNF-α (**e**), IL-12 (**f**), IL-4 (**g**), IL-10 (**h**) and IFN-γ/IL-10 ratio (**i**)*.* The *x*-axis represents the control group (C), Leishmune® and LdCen^−/−^ vaccines. The connecting lines between the bars indicate significant differences (*P* < 0.05)

Since the CD8^+^ T-lymphocytes act as effector cells to control intracellular pathogens, this T-cell subset was included in the co-culture system to analyze the induction of memory. LdCen^−/−^ (*P* = 0.010) and Leishmune® (*P* = 0.009) groups presented significant reduction in the number of amastigotes in macrophages co-cultured with CD8^+^ T cells as compared to the control group (Fig. 2c). All analyzed pro-inflammatory cytokines (IFN-γ, TNF-α, and IL-12; Fig. 2d-f) presented high levels in co-cultures of both LdCen^−/−^ and Leishmune® groups as compared to the control group. In contrast, LdCen^−/−^ (*P* = 0.018) and Leishmune® (*P* = 0.034) groups displayed lower levels of IL-4 (Fig. 2g) and IL-10 (Fig. 2h) as compared to the control group. Interestingly, LdCen^−/−^ (*P* = 0.006) and Leishmune® (*P* = 0.012) groups showed increased IFN-γ/IL-10 ratio, in addition to LdCen^−/−^ immunization eliciting the highest (*P* = 0.008) cytokine ratio (Fig. 2i).

### The co-culture system using macrophages plus CD4^+^:CD8^+^ T-cells simultaneously revealed high anti-leishmanicidal activity, increased levels of pro-inflammatory cytokines, and a reduction of IL-4 and IL-10 in both LdCen^−/−^ and Leishmune® groups

**Fig. 3:**
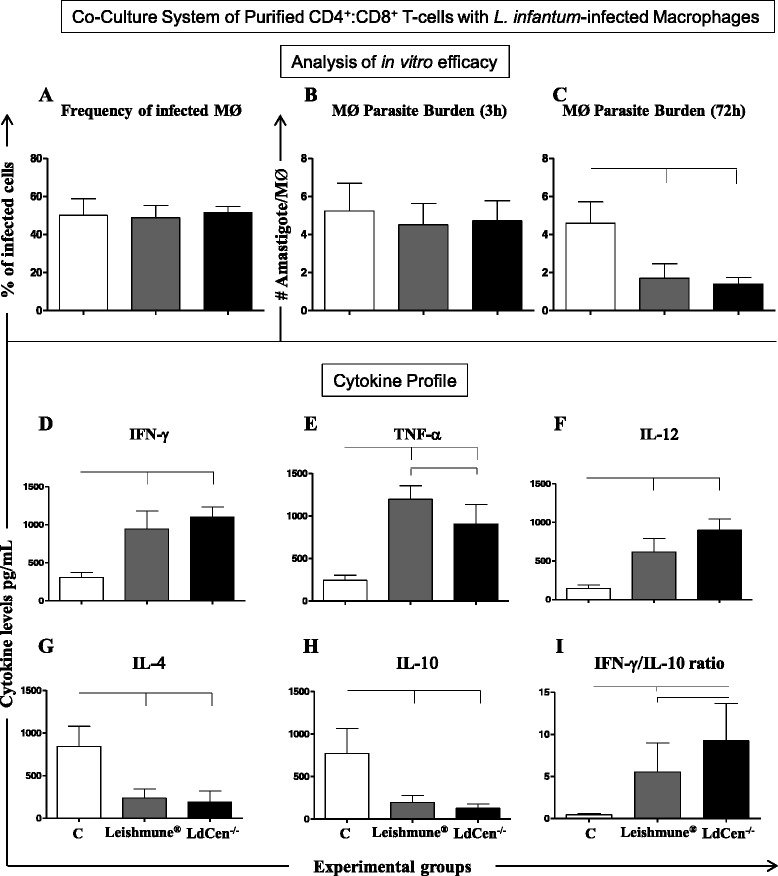
Co-culture system using *L. infantum* infected macrophages (MØ) and CD4^+^: CD8^+^ T-cells, 24 months after experimental challenge with *L. infantum*. Control group (*n* = 6 dogs) and Leishmune® (*n* = 5 dogs) received three doses of PBS or vaccine, respectively, at 21 day intervals; LdCen^−/−^ group (*n* = 6 dogs) received 10^7^ LdCen^−/−^ stationary phase promastigotes in single-dose. **a** Infection rate (%) of macrophages after 3 h of in vitro infection with *L. infantum* (5 × 10^6^ promastigotes; ratio ten parasites: 1 MØ). **b** Number of amastigote forms by macrophages after 3 h of in vitro infection with *L. infantum*. **c** Number of amastigote forms by macrophages after 72 h of in vitro infection with *L. infantum*. The cytokine profile (pg/mL) was analyzed in supernatant from *L. infantum* infected macrophages (MØ) co-cultured with CD4^+^:CD8^+^ T-cells for IFN-γ (**d**), TNF-α (**e**), IL-12 (**f**), IL-4 (**g**), IL-10 (**h**) and IFN-γ/IL-10 ratio (**i**)*.* The *x*-axis represents the control group (C), Leishmune® and LdCen^−/−^ vaccines. The connecting lines between the bars indicate significant differences (*P* < 0.05)

The co-culture system using macrophages associated with CD4^+^: CD8^+^ T-cells simultaneously was employed to analyze the addictive effect of adaptive immune response in the context of T-cells subsets. In this sense, LdCen^−/−^ (*P* = 0.001) and Leishmune® (*P* = 0.007) immunizations induced a reduction in the parasite burden at 72 h post infection with *L. infantum* as compared with the control group (Fig. 3c). The cytokine analysis revealed high levels of IFN-γ (Fig. 3d), TNF-α (Fig. 3e) and IL-12 (Fig. 3f) in both LdCen^−/−^ and Leishmune® groups when compared to control group. Moreover, the Leishmune® immunization was able to induce high amounts of TNF-α as compared to the LdCen^−/−^ group (*P* = 0.042). Furthermore, both analyzed vaccines showed low levels of IL-4 (Fig. 3d) and IL-10 (Fig. 3h). Similar to co-cultures using purified CD4^+^ or CD8^+^ T-cells, the co-culture system using CD4^+^:CD8^+^ T-cells simultaneously was able to identify in LdCen^−/−^ and Leishmune® groups a high (*P* = 0.016) IFN-γ/IL-10 ratio, plus the LdCen^−/−^ group displayed the highest cytokine ratio (Fig. 2i).

## Discussion

This study applied previously standardized protocols [12, 13] of co-culture systems using *L. infantum*-infected macrophages and purified CD4^+^ or CD8^+^ T-cell subsets from immunized dogs, 24 months after experimental challenge with a virulent strain of *L. infantum*. This method was previously developed to analyze vaccine candidates against canine visceral leishmaniasis [12, 13]. LdCen^−/−^ and Leishmune® vaccines were used as they have demonstrated a strong immunogenicity [10, 11, 14–18].

In this sense, it has been described that immunization with LdCen^−/−^ in dogs elicited high levels of serum immunoglobulins, a lymphoproliferative response, and T-cell activation, which increased production of Th1 cytokines and decreased secretion of Th2 cytokines [14]. Furthermore, it was also shown that LdCen^−/−^ and Leishmune® immunizations resulted in significant protection, as revealed by the low parasite burden in the bone marrow at 18 months and even 24 months post challenge [11, 14]. The parasite burden level observed in the LdCen^−/−^ and Leishmune® immunized group is comparable to that typically observed in asymptomatic dogs, which are PCR positive [19], suggesting a robust degree of protection. These data support the application of LdCen^−/−^ and Leishmune® immunizations in the current evaluation as reference vaccines to validate the proposed co-culture system.

Most studies involving co-culture of macrophages and lymphocytes are empirically developed, using total lymphocytes, and do not reflect the cytokine levels of the T-cells. The method described in this study applied a new, more accurate approach that yields useful laboratory parameters to evaluate the ability of canine immune cells to control in vitro *L. infantum* infection as a biomarker to identify the immunogenicity mechanism.

Traditional immunogenicity studies in vaccines against CVL, such as Leishvacin or Leishmune®, have described the biomarkers regarding increased percentage of T lymphocytes producing IFN-γ, especially CD4^+^ T-cells [15, 16]. Moreover, LiESAp-MDP vaccine elicited protection that was correlated with the early establishment of a long-lasting, predominantly Th1-type cellular immune response relationship to enhanced in vitro leishmanicidal activity of macrophages in response to higher IFN-γ production by T-cells [20]. Recently, a study evaluating the LiESP/QA-21 (CaniLeish®) vaccine demonstrated strong cell-mediated immune responses against the parasite despite a virulent challenge associated with a lower probability of developing active infections [21]. Furthermore, tests for microbicidal activity of macrophages cultured with autologous total lymphocytes showed that the vaccinated group inhibited twice the parasite replication in comparison with the control group [21]. However, the ideal method for identifying the protective mechanisms induced by vaccine against canine visceral leishmaniasis would analyze the effector immune response. In this sense, the leishmanicidal effect could be evaluated as an effector mechanism induced by T-cells subsets co-cultured with *L. infantum* infected macrophages.

Our data demonstrated that the three co-culture systems (*L. infantum* infected macrophages with purified CD4^+^ or CD8^+^ T-cells or CD4^+^:CD8^+^ T-cells simultaneously) from LdCen^−/−^ and Leishmune® were able to identify a significant reduction in the parasitic burden. Noteworthy is the fact that the co-culture system containing a purified CD4^+^ T-cell subset was able to recognize a differential capacity of LdCen^−/−^ vaccine to induce a strong in vitro leishmanicidal control when compared to Leishmune® immunization. In fact, the strong leishmanicidal ability could be associated with differential IFN-γ production in addition to a higher IFN-γ/IL-10 ratio, as presented by the co-culture system containing the purified CD4^+^ T-cell subset. In previous studies regarding asymptomatic *L. infantum-*infected dogs, co-cultures with CD8^+^ T-cells and infected-macrophages were able to produce high levels of IFN-γ, whereas cells from symptomatic dogs did not [22].

Type 1 cytokine analysis has been considered a prerequisite for immunogenicity analyses before and after experimental challenge with *L. infantum* in clinical anti-CVL vaccine trials [6, 12]. In fact, the co-culture system proposed herein was able to identify the type 1 cytokine pattern, in agreement with an earlier studies for both LdCen^−/−^ and Leishmune® immunizations [11, 14]. Moreover, Leishmune® immunization elicited high TNF-α production in a co-cultured system with CD4^+^:CD8^+^ T-cells simultaneously, but without effect in producing differential parasitism control when compared to LdCen^−/−^. Interestingly, IFN-γ, but not TNF-α levels, was associated with immunogenicity and protection in dogs immunized with LBSap vaccine [23]. Furthermore, it was reported that dogs immunized with purified excreted-secreted antigens of *Leishmania infantum* presented high levels of IFN-γ in co-culture with *L. infantum* infected macrophages and total lymphocytes, inducing a microbicidal effect [24, 25].

Considering the previous studies describing strong immunogenicity elicited by LdCen^−/−^ and Leishmune® immunizations, this study applied a new approach based on a co-culture system using *L. infantum* infected macrophages and purified T-cell subsets after vaccination and experimental *L. infantum* challenge. Data from this study indicated that the co-culture system was able to identify the immunogenicity mechanism induced by LdCen^−/−^ and Leishmune® vaccination in dogs, in addition to associating an effector mechanism (in vitro leishmanicidal control by *L. infantum* infected macrophages) regarding an induction of T-cell memory.

## Conclusions

The low levels of type 2 cytokines (IL-4 and IL-4), high amounts of type 1 cytokines (IFN-γ, TNF-α and IL-12), and increased IFN-γ/IL-10 ratio were associated with a leishmanicidal effect of *L. infantum* infected macrophages, using co-culture systems with CD4^+^ or CD8^+^ or CD4^+^: CD8^+^ T-cells simultaneously. This strategy was able to validate the distinct conditions of the analyzed co-culture systems. Thus, taken together, the data point to the in vitro co-culture systems providing a rapid assay to test and monitor the immunogenicity and efficacy of *Leishmania* vaccines in dogs.
